# Comparison of the effects of different infant formulas on the growth and development and intestinal flora of infants

**DOI:** 10.1002/fsn3.3149

**Published:** 2022-12-07

**Authors:** Ning Yin, Xinran Liu, Xiaoxuan Zhang, Jing Wen, Huijuan Ma, Xueqian Yin, Craig‐Kui Xie, Yanmei Hou, Junbo Wang

**Affiliations:** ^1^ Department of Nutrition and Food Hygiene, School of Public Health Peking University Beijing China; ^2^ Ausnutria Hyproca Nutrition Co., Ltd. Changsha China; ^3^ Beijing Key Laboratory of Toxicological Research and Risk Assessment for Food Safety Peking University Beijing China

**Keywords:** development, formula, growth, infant, intestinal flora

## Abstract

The purpose of this study was to compare the effects of different infant formulas on the growth and development, sleep, allergy symptoms, and intestinal flora of infants. A total of 428 infants participated in the study. Breastfeeding (BF) was used as the control, and the remaining subjects were randomly assigned to the full goat milk protein formula group (FGM), partial goat milk protein formula group (PGM), and cow milk formula group (M). During the 6‐month feeding experiment, data on the growth, sleep, allergy symptoms, and intestinal flora of infants were collected using questionnaires, anthropometric measurements, and biochemical examinations. In general, the basic information of the participants was consistent among the groups. There were no differences in infant weight, length, or head circumference among the groups (*p* > .05). The sleep time of infants in the formula‐fed groups was longer than that of the breastfeeding group at baseline (*p* < .05), but there were no differences at mid‐term or outcome (*p* > .05). The incidence of allergic symptoms continued to decrease, and the total scores of allergic symptoms did not differ among the groups (*p* > .05). The relative abundance of intestinal *Bifidobacteriaceae* in the PGM group was lower than that in the other groups (*p* < .05). There was no difference in the *β*‐diversity of intestinal flora between formula‐fed and breastfed infants (*p* > .05). There were strong correlations in the composition of the main intestinal flora at the family level between the formula and breastfeeding groups. This study showed that within 6 months of feeding, there were no significant differences in the growth and development, allergic symptoms, or intestinal flora of the infants among the groups.

## Graphical abstract

Early in life (0–3 months of age) breastfed infants probably had better growth and development than either artificial or mixed feeding, but compared with breastfeeding, several formula feedings were able to meet the growth and developmental needs of infants within 6 months of feeding; there were no significant differences in the growth and development, allergic symptoms, and intestinal flora of the infants among the groups.

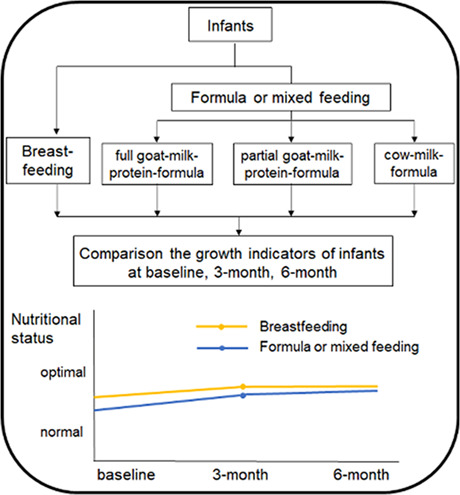

## 1 INTRODUCTION

Feeding patterns are the main factors affecting the nutritional status of newborns and infants, and their nutritional status further affects their growth and development. Breastfeeding is an unequal way of providing ideal food for the healthy growth and development of infants (American Academy of Pediatrics (AAP), 2012). As a global public health recommendation, infants should be exclusively breastfed for the first 6 months of life to achieve optimal growth, development, and health (Kramer & Kakuma, 2012).

Lack of breastfeeding, especially in the first half of life, is an important risk factor for infant and childhood morbidities and mortality (World Health Organization, 2002). However, most newborns and infants under 6 months of age in China cannot be exclusively breastfed (Chang & Wang, 2016; Niu‐niu et al., 2020; Shu‐ge et al., 2017). According to the 2013 Report on Nutrition and Monitoring of Chinese Residents, the rate of exclusive breastfeeding of infants under 6 months was 20.8%, and the rate of continuing breastfeeding aged 20–23 months was only 7.0% (Chang & Wang, 2016). Li et al. found that the rates of exclusive breastfeeding within 3 months and 6 months were 34.4% and 14.1%, and the rates of basic exclusive breastfeeding were 61.6% and 55.6%, respectively (Niu‐niu et al., 2020). These data show that most infants could not be breastfed. For these newborns and infants, it is important to choose the appropriate formulas for their growth, development, and health needs.

Prior to this, most studies have compared the effects of goat milk powder and cow milk powder on the growth and development of infants or animals (Grant et al., 2005; Xu, Wang, et al., 2015; Xu, Wei, et al., 2015; Zhou et al., 2014). The results of these studies are also controversial. In this study, breastfeeding was used as a control, and a multicenter trial was conducted to compare the differences in the growth and development and intestinal flora of infants between different formulas and breastfeeding and to determine whether the formula could meet the needs of infant growth and development.

## 2 MATERIALS AND METHODS

### 2.1 Study design and participants

Three maternal and child healthcare hospitals in Shunyi, Beijing, Shiyan, Hubei, and Hebi, Henan, were selected as research sites in 2016. When conducting physical examinations for pregnant and lying‐in women, the local maternal and child healthcare doctors issued recruitment advertisements to them, explained the experiment, and signed the informed consent form with those who were willing to participate and met the inclusion criteria. A total of 428 infants participated.

During the grouping process, to respect the choice of the infant's family and improve compliance, the original feeding method of the infants was not changed: infants who were breastfed would continue to maintain breastfeeding and enter the breastfed group (BF) as the control; infants who had already started artificial feeding or mixed feeding were randomly divided into three groups: full goat milk protein formula group (FGM, whey protein is goat milk source), partial goat milk protein formula group (PGM, whey protein is a mixed source of goat milk and cow milk), and cow milk formula group (M) using the random number table method. The randomization table was created in advance using Microsoft Excel 2016. The serial numbers were determined according to the order of entry, and the intervention measures of the corresponding groups of the numbers were taken. Subsequently, the grouping information was sent to the implementer—local maternal and child healthcare hospital.

Groups BF, FGM, PGM, and M were fed breast milk, commercial goat formula, non‐whole goat milk formula, and cow milk formula, respectively, according to the normal needs and feeding habits of infants under the guidance of maternal and child healthcare doctors (see Appendix A for the nutritional ingredients of each formula). The observation period was 6 months. Infants were investigated for growth, development, sleep, and allergy symptoms at enrollment and at 3 months (mid‐term) and 6 months (outcome), and the intestinal flora of infants were investigated at enrollment and outcome.

### 2.2 Inclusion and exclusion criteria

The inclusion criteria were as follows: infants aged 0 ~ 3 months, no serious genetic diseases or mental disorders, congenital defects, living in the local area after delivery, taking care of the baby by themselves or participating in the care of the baby as a primary member, and willingness to cooperate in the study.

The exclusion criteria were as follows: multiple births; vaginal midwifery (forceps and fetal suction); low birth weight (<2500 g); divorced or widowed during pregnancy or after childbirth; mothers who became pregnant again while breastfeeding; infants with allergies to medicines and/or foods; participation in other clinical trials within 4 weeks; other diseases or factors affecting absorption, distribution, metabolism, and excretion; and those who did not meet the inclusion criteria and did not eat the tested samples as prescribed, could not determine the curative effect, or lacked information affecting the judgment of curative effect.

### 2.3 ETHICS STATEMENT

This study was approved by the Peking University Medical Ethics Committee (approval no: IRB00001052‐16037), and the mother or other guardians of each participant fully understood the relevant content of this study and signed an informed consent form.

### 2.4 Sample size

This study was a parallel controlled trial. Using the difference test, the sample size calculation used the change in body weight (the primary outcome variable) as the indicator. Taking the 8‐month‐old weight of infants in the breastfeeding group in “Effects of Feeding Methods on the Physical Development Trajectory of Infants” as the population mean and standard deviation (SD) of the breastfeeding group (Xiangqing et al., 2011), the 7‐month‐old weight data of infants in the M, PGM, and FGM groups in the literature report (Yibin et al., 2013) were used to set the population mean and SD. It was supposed that the probability of a type I error is 0.05, and the probability of a type II error is 0.9. The sample size was estimated to be 75 for each group using PASS (version 11, NCSS, UT, USA) one‐way analysis of variance among multiple groups. Considering the loss‐to‐follow‐up rate of 30%, the sample size for each group was 107 and the total sample size was 428.

**FIGURE 1:**
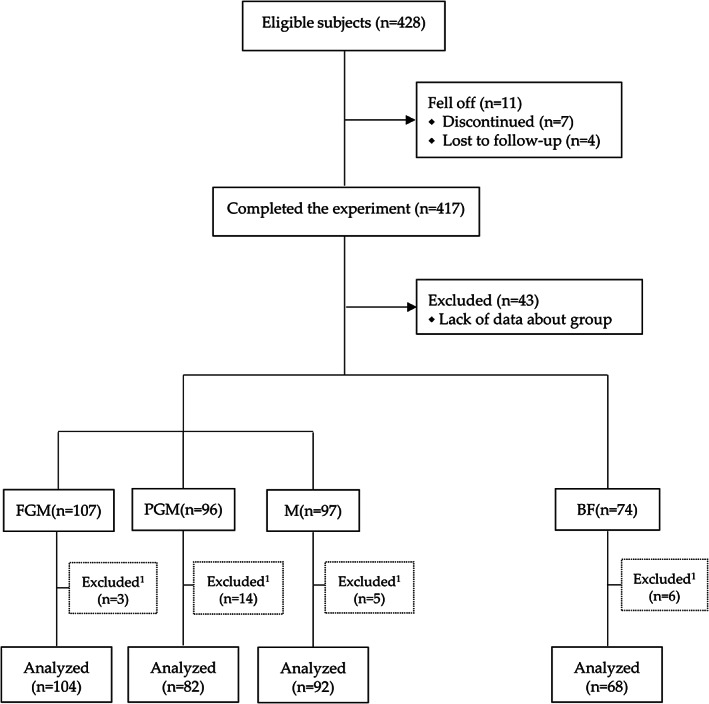
Flow diagram of participants throughout the study, from enrollment to study completion. FGM, Full goat milk protein formula group; PGM, Partial goat milk protein formula group; M, Cow milk formula group; BF, Breastfeeding. ^1^, Because of incomplete questionnaire information or obvious logical errors

A total of 428 infants were included in this study. Of the 417 participants who completed the experiment, 11 fell off. The reasons for falling off were as follows: 4 were lost to follow‐up and 7 quit. Due to a lack of data about the group, incomplete questionnaire information, or obvious logical errors in part of the questionnaire data, 71 infants could not be included in the final analysis, and a total of 346 infants were included in the final analysis (Figure 1).

### 2.5 Test substances

In this study, infants in the artificial feeding groups received formulas sold at the market and qualified. Formulas were entrusted to the local maternal and child healthcare hospital for regular verification and free distribution. The frequency may vary for each infant depending on the specific situation of on‐demand feeding.

### 2.6 Definitions

#### 2.6.1 Growth velocity

The increase amounts in body weight/length/head circumference of a child over a specified period (e.g., a month or year). In this study, the growth velocities of body weight, length, and head circumference were calculated in 3‐month increments.

#### 2.6.2 Nutritional status

The nutritional status of the infants was determined according to the *Z* score standard recommended by the WHO. WAZ < −2, low weight; WAZ >2, overweight; WHZ < −2, wasting; WHZ >2, obesity; and HAZ < −2, growth retardation. The presence of one or more of these conditions indicates malnutrition.

#### 2.6.3 Total sleep time

Cumulative value of each sleep duration over a 24‐hour day.

#### 2.6.4 Allergy symptom score

The main symptoms of infant allergies include crying, vomiting, eczema, and other skin and respiratory symptoms. Allergic reactions in severity can range from mild to severe.

The severity of each allergy symptom was scored according to the infant's weight, and the total result was the allergy symptom score.

### 2.7 Data collection

#### 2.7.1 General information

The general information of participants' mother and their family were collected using questionnaires, including basic information on parents, such as age, education, occupation, and per capita monthly household income, pregnancy, delivery history, and basic information on the participants, including mode of delivery, pregnancy complications, birth weight and length, sex, feeding method, etc.

#### 2.7.2 Anthropometric measurements

Physical development indicators of infants, including weight, body length, and head circumference, were measured by professional physicians. Weight was measured with the maximum load 50 kg lever scale for children, in kilograms (kg) as a unit; body length was measured by supine position in centimeters (cm) as a unit; and head circumference was measured by a tape measure around the eyebrow arch and inion for a circle; these results were accurate to 0.1 kg/cm. All anthropometric growth data were converted to *Z* score using WHO Child Growth Standards.

#### 2.7.3 Sleep and allergy symptoms

The sleep and allergy symptom indices of infants were collected through outpatient follow‐up, telephone interviews, and door‐to‐door visits. According to the Sleep Hygiene Guidelines for Children 0–5 years of age, the Brief Infant Sleep Questionnaire (BISQ) for children 0–2 years of age (Appendix B) was used to assess infant sleep (579–2017, n.d.; Avi Sadeh, 2004). This questionnaire has been widely used in many studies in many countries (Kohyama et al., 2011; Nunes et al., 2012; Sadeh et al., 2009; Teng et al., 2012). According to cow milk‐related symptom score (CoMiSS), it was established by Vandenplas et al. (2015) to evaluate the occurrence of allergic symptoms in infants (Appendix C). The CoMiSS was verified, and published data using the CoMiSS in clinical trials showed that the predictive value of the tool was 80% (Salvatore et al., 2019; Vandenplas et al., 2018).

### 2.8 Fecal collection and intestinal flora detection

Fresh feces were picked up with a sterile swab by the investigators, placed into sterile sampling cups immediately (placed in an ice box), and stored in a − 80°C refrigerator within 2 h. The samples were sent to Beijing by whole cold‐chain transport, and they were kept at −80°C before being sent to the laboratory for testing.

DNA extraction and quality inspection: A UV microspectrophotometer (NanoDrop 2000, Thermo Fisher Scientific, Inc) was used for total DNA extraction, and 1% agarose gel electrophoresis was used to analyze the purity and integrity of DNA and to quantify the DNA concentration.

16 S rDNA sequencing: The region for 16 S rDNA amplification in the V3‐V4 region was selected. 341F and 806R were used as universal primers, and the index sequence and connector sequence suitable for Illumina MiSeq PE250 sequencing were added to the 5′ end of the two primers to complete the design of specific primers. The Illumina platform was used to obtain paired‐end data of PE250, and a long sequence was obtained by splicing to carry out 16 S analysis.

### 2.9 Stopping rules for the trial and withdrawal criteria

1. If serious safety problems were encountered during the test, the test should be promptly terminated.
2. Significant errors in the prescribed program were found in the test, which made it difficult to evaluate the intervention effect. Alternatively, in the implementation of serious deviations, the intervention effect was difficult to evaluate.
3. The applicant requests termination of the experiment or the administrative department requests termination of the experiment.
4. Subjects could opt out of the study at any stage.

### 2.10 Statistical analysis

SPSS (version 23.0, IBM, Armonk, NY, USA) was used for statistical analysis of the data, the composition of counting data was described, and the Chi‐square test was used for analysis. The measurement data were compared using one‐way analysis of variance (ANOVA) on the premise of normality and homogeneity of variance, and the LSD method was used for pairwise comparison. If normality was met but variances were not uniform, the Welch test method was used to compare the differences among groups, and Dunnett's test was used for pairwise comparisons. If normality was not satisfied, the nonparametric test was used to compare the differences among groups. A two‐sided *p* < .05 was considered statistically significant.

## 3 RESULTS

### 3.1 General information

The analysis of some basic information before enrollment showed that the overall information distribution of infants, their parents, and their families in each group was almost balanced (Table 1).

**TABLE 1:** General information about the participants

	Total	BF	FGM	PGM	M	F/χ2	p
Parents' situation							
Mother's age (years)	30.3 ± 4.8	29.0 ± 4.9	30.1 ± 4.0	30.9 ± 4.9^a^	31.1 ± 5.2^a^	*F* = 2.809	.040
BMI of mother (kg/m^2^)	23.8 ± 3.1	23.4 ± 2.6	24.0 ± 3.1	23.4 ± 3.5	24.2 ± 3.2	*F* = 1.183	.316
Father's age (years)	31.7 ± 5.3	30.7 ± 5.7	31.5 ± 4.5	32.2 ± 5.4	32.3 ± 5.7	*F* = 1.408	.240
BMI of father (kg/m^2^)	24.0 ± 3.3	23.5 ± 3.4	24.2 ± 3.4	24.4 ± 3.4	23.9 ± 3.0	*F* = 1.073	.361
Family situation							
Monthly household food consumption (CNY)							
0‐	45 (13.5)	5 (8.2)	14 (13.5)	12 (15.0)	14 (15.9)	*χ* ^2^ = 4.394	.623
1000‐	191 (57.4)	34 (55.7)	57 (54.8)	47 (58.8)	53 (60.2)		
2000‐	97 (29.1)	22 (36.1)	33 (31.7)	21 (26.2)	21 (23.9)		
Infant situation							
Age of enrollment (days)	48.8 ± 23.7	51.5 ± 18.6	43.3 ± 23.3	53.4 ± 26.5^b^	48.9 ± 23.9	*F* = 3.301	.021
Birth weight (kg)	3.38 ± 0.42	3.40 ± 0.39	3.39 ± 0.46	3.35 ± 0.40	3.40 ± 0.40	*F* = 0.262	.853
Birth length (cm)	50.3 ± 1.2	50.1 ± 1.2	50.3 ± 1.3	50.3 ± 1.3	50.3 ± 1.1	*F* = 0.294	.830
Head circumference (cm)	34.1 ± 1.4	33.9 ± 0.9	34.2 ± 1.2	34.1 ± 1.4	34.1 ± 1.7	*F* = 0.188	.904
Chest circumference (cm)	33.7 ± 1.5	33.6 ± 1.2	33.8 ± 1.3	33.4 ± 1.4	33.8 ± 1.8	*F* = 0.846	.470
WAZ	0.23 ± 1.31	0.24 ± 1.02	0.27 ± 1.26	0.26 ± 1.56	0.14 ± 1.37	*F* = 0.164	.920
WLZ	0.53 ± 1.49	0.77 ± 1.05	0.37 ± 1.23	0.54 ± 1.80	0.53 ± 1.74	*F* = 0.943	.420
LAZ	−0.12 ± 1.68	−0.26 ± 1.17	0.12 ± 1.50	−0.21 ± 2.03	−0.22 ± 1.63	*F* = 1.066	.364
Sex							
Male	172 (49.9)	29 (42.6)	50 (48.1)	47 (57.3)	46 (50.5)	*χ* ^2^ = 3.389	.335
Modes of delivery							
Natural delivery	142 (41.2)	36 (52.9)	35 (34.0)	37 (45.1)	34 (37.0)	*χ* ^2^ = 8.000	.238
Artificial assisted delivery	29 (8.4)	6 (8.8)	10 (9.7)	6 (7.3)	7 (7.6)		
Cesarean section	174 (50.4)	26 (38.2)	58 (56.3)	39 (47.6)	51 (55.4)		

*Note*: Continuous variables were expressed as the mean ± *SD* and categorical variables were expressed as frequency and percent.

Abbreviations: BF, breastfeeding; CNY, Chinese Yuan; F, Analysis of variance; FGM, full goat milk protein formula group; LAZ, Length for age *Z* score; M, cow milk formula group; PGM, partial goat milk protein formula group; WAZ, Weight for age *Z* score; WLZ, Weight for length *Z* score; *χ* ^2^, Chi‐square test.

^a^ Compared with the BF group, there was a significant difference.

^b^ Compared with the FGM group, there was a significant difference.

### 3.2 Growth and development

**FIGURE 2:**
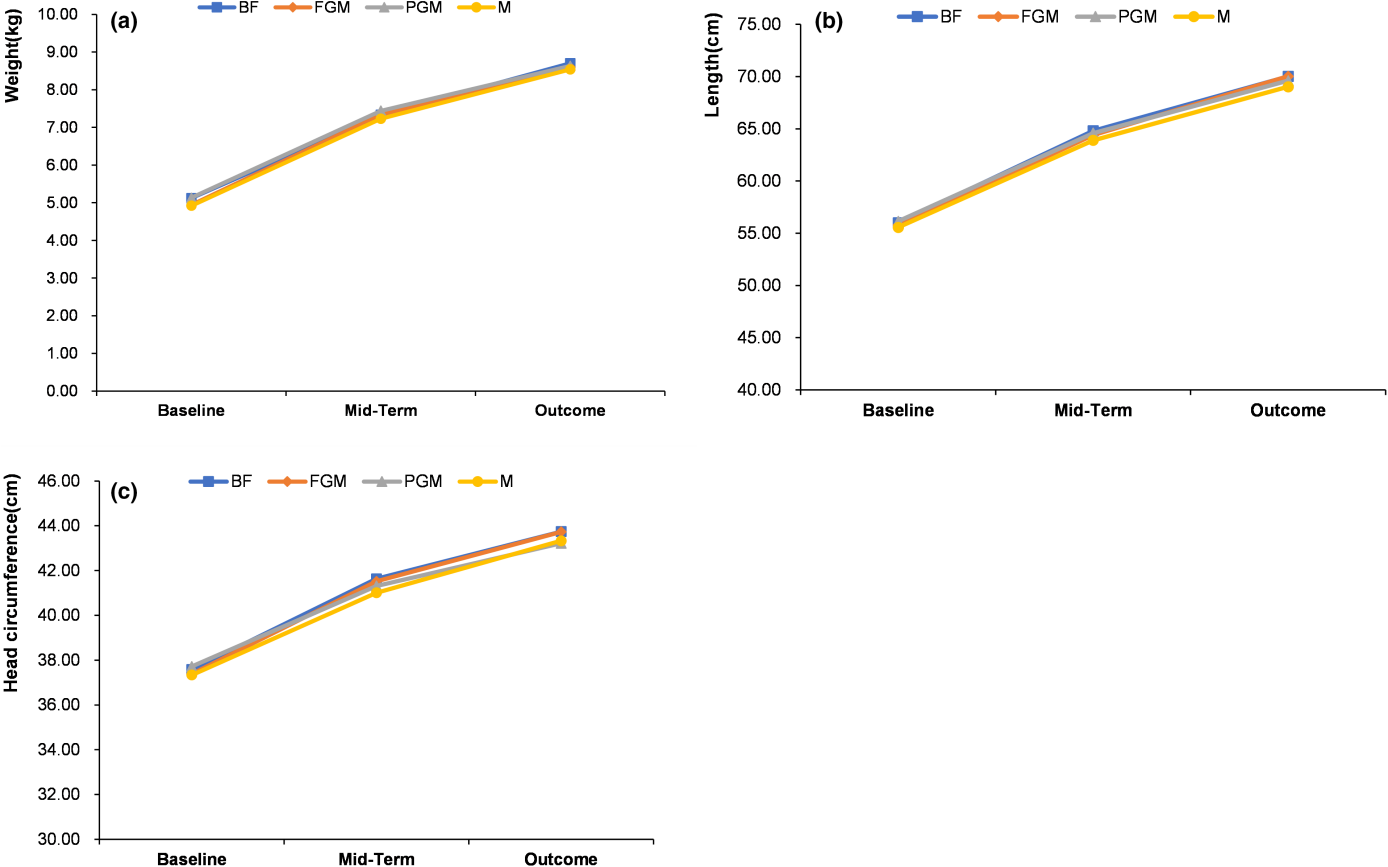
Effects of different formulas on the growth and development of infants: (a) weight; (b) length; and (c) head circumference.

At baseline, mid‐term, and outcome, there were no significant differences in the length, weight, or head circumference of the infants among the different feeding groups (*p* > .05). There was also no difference in the gains in length, weight, or head circumference among all groups (*p* > .05), as shown in Figure 2.

**FIGURE 3:**
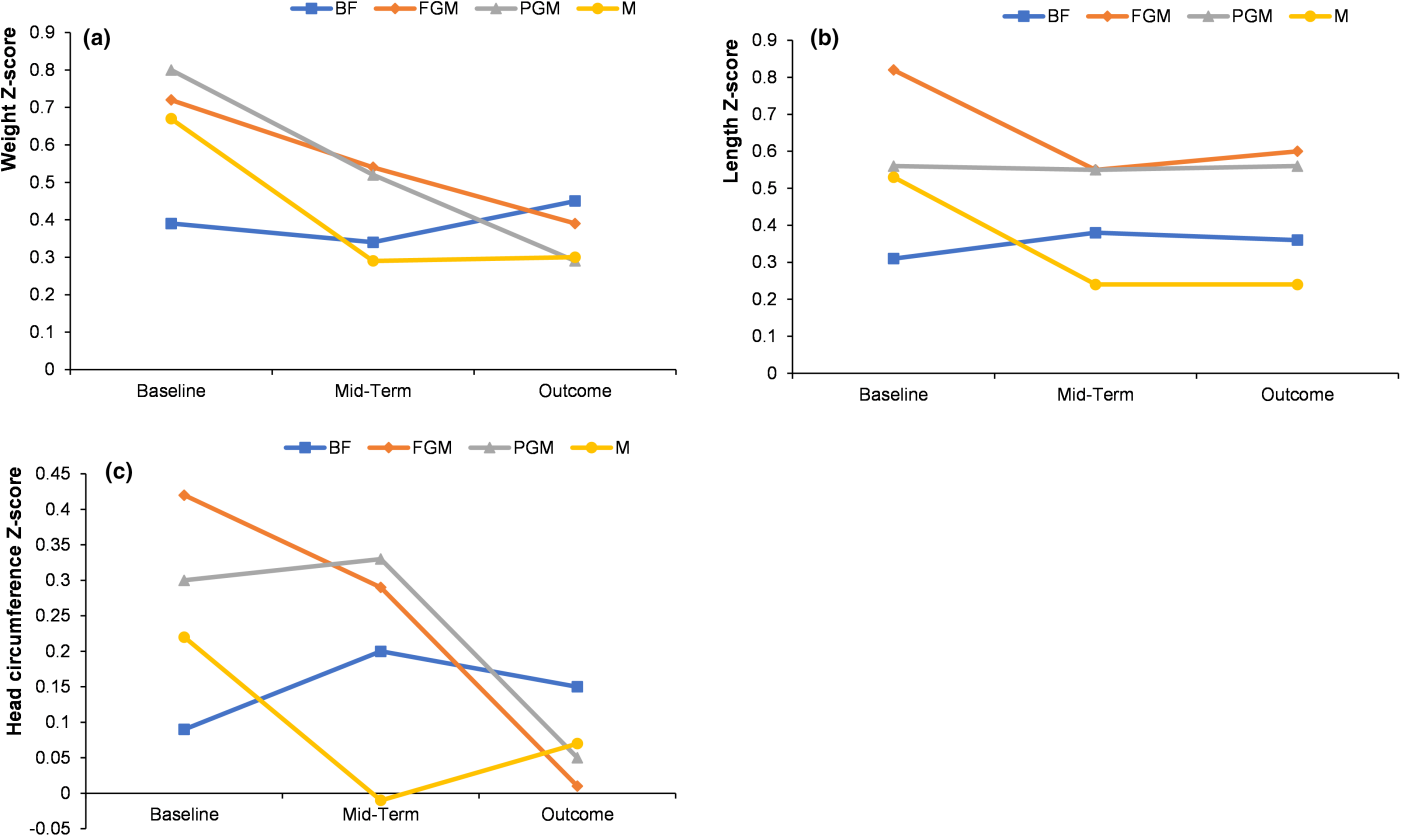
Effects of different formulas on the growth and development of infants: (a) weight *Z* score; (b) length *Z* scores; and (c) head circumference *Z* score.

The infants' weight, length, and head circumference *Z* score among the different feeding groups were all within 1 SD of WHO child growth standards during the entire experiment (Figure 3).

According to the Child Growth and Development Standards (2006) recommended by the WHO, weight for age *Z* score (WAZ), weight for length *Z* score (WLZ), BMI *Z* score, weight velocity *Z* score, length for age *Z* score (LAZ), length velocity *Z* score, head circumference for age *Z* score (HCAZ), and head circumference velocity *Z* score were calculated and compared among different feeding groups, but there was little significant difference among the groups (Tables 2 and 3).

**TABLE 2:** Effects of different feeding methods on *Z* scores for the weight of infants (Mean ± *SD*)

Group	Weight for age *Z* score (WAZ)			Weight for length *Z* score (WLZ)			BMI *Z* score			Weight velocity *Z* score	
	Baseline	Mid‐term	Outcome	Baseline	Mid‐term	Outcome	Baseline	Mid‐term	Outcome	3 months	6 months
BF	0.24 ± 1.02	0.39 ± 1.11	0.61 ± 1.05	0.77 ± 1.05	0.44 ± 1.18	0.61 ± 1.19	0.46 ± 1.05	0.30 ± 1.12	0.49 ± 1.20	0.30 ± 1.58	0.52 ± 1.38
FGM	0.27 ± 1.26	0.55 ± 1.01	0.56 ± 0.86	0.37 ± 1.23	0.62 ± 1.37	0.50 ± 1.10	0.05 ± 1.29	0.50 ± 1.22	0.36 ± 1.08	0.47 ± 1.38	0.42 ± 1.09
PGM	0.26 ± 1.56	0.50 ± 0.95	0.48 ± 1.01	0.54 ± 1.80	0.79 ± 1.65	0.57 ± 1.35	0.19 ± 1.77	0.63 ± 1.40	0.45 ± 1.41	0.33 ± 1.48	0.36 ± 1.42
M	0.14 ± 1.37	0.27 ± 0.87	0.39 ± 0.89	0.53 ± 1.74	0.58 ± 1.27	0.66 ± 1.15	0.19 ± 1.73	0.45 ± 1.15	0.53 ± 1.12	0.09 ± 1.45	0.28 ± 1.34
*F*	0.164	1.316	0.730	0.943	0.672	0.245	1.006	0.747	0.281	0.952	0.411
*p*	0.920	0.269	0.535	0.420	0.570	0.865	0.391	0.525	0.839	0.416	0.745

**TABLE 3:** Effects of different feeding methods on *Z* scores for length and head circumference of infants (Mean ± *SD*)

Group	Length for age *Z* score (LAZ)			Length velocity *Z* score		Head circumference for age *Z* score (HCAZ)			Head circumference velocity *Z* score	
	Baseline	Mid‐term	Outcome	3 months	6 months	Baseline	Mid‐term	Outcome	3 months	6 months
BF	−0.26 ± 1.17	0.30 ± 1.39	0.38 ± 1.42	0.83 ± 2.37	0.72 ± 2.01	−0.39 ± 1.09	−0.04 ± 1.04	−0.01 ± 1.12	0.54 ± 2.47	2.00 ± 2.61
FGM	0.12 ± 1.50	0.29 ± 1.33	0.54 ± 1.49	0.26 ± 2.29	0.77 ± 2.19	−0.15 ± 1.60	0.02 ± 1.24	0.00 ± 1.18	0.33 ± 3.02	1.59 ± 2.81
PGM	−0.21 ± 2.03	0.08 ± 1.58	0.12 ± 1.68	0.42 ± 3.00	0.62 ± 2.65	−0.32 ± 1.76	−0.33 ± 1.49	−0.43 ± 1.52	0.03 ± 3.09	1.77 ± 3.55
M	−0.22 ± 1.63	−0.15 ± 1.21	−0.04 ± 1.38	0.07 ± 2.87	0.15 ± 2.29	−0.45 ± 1.51	−0.53 ± 1.39^a^	−0.26 ± 1.95	0.07 ± 3.24	2.29 ± 5.15
*F*	1.066	1.879	2.439	0.961	1.107	0.564	2.996	1.224	0.358	0.373
*p*	0.364	0.133	0.065	0.412	0.347	0.639	0.031	0.302	0.783	0.772

^a^ Compared with the BF group, there was a significant difference.

### 3.3 Nutritional status

**FIGURE 4:**
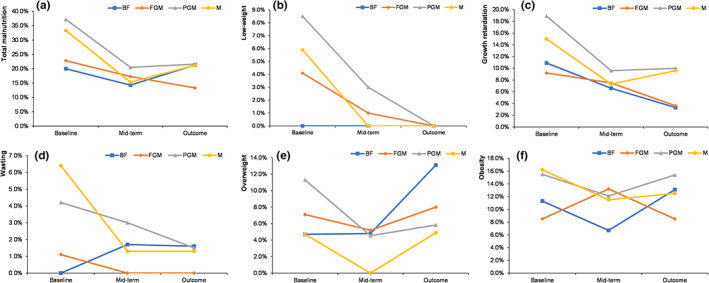
Effects of different formulas on the nutritional status of infants: (a) total malnutrition; (b) low weight; (c) growth retardation; (d) wasting; (e) overweight; and (f) obesity.

Figure 4 shows the results of infant nutritional status at baseline, mid‐term, and outcome in this study. At baseline, the prevalence rates of low weight, wasting, overweight, obesity, growth retardation, and total malnutrition in the formula feeding groups were higher than those in the breastfeeding group, and the prevalence of total malnutrition in the PGM group was higher than that of the BF group (*p* < .05).

After feeding for 3 months, compared with the baseline, the prevalence of all types of malnutrition in all groups showed a decreasing trend. After feeding for 6 months, the prevalence of low weight and wasting in each group continued to decrease, but the prevalence of overweight, obesity, and total malnutrition increased, and there were no differences among the different feeding groups (*p* > .05).

### 3.4 Sleep

**FIGURE 5:**
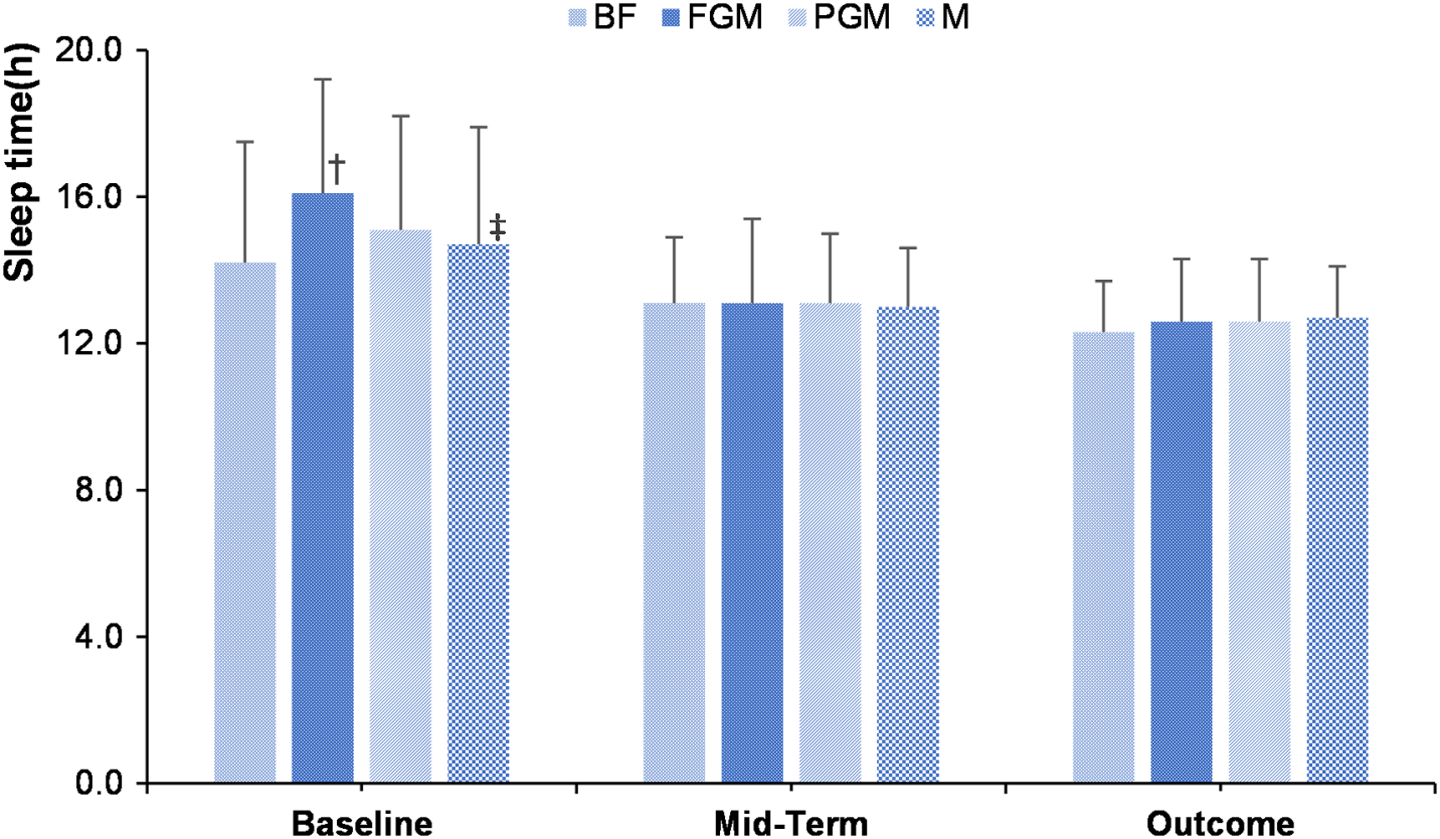
Effects of different formulas on sleep time of infants; †, Compared with the BF group, there was a significant difference; ‡, Compared with the FGM group, there was a significant difference

Figure 5 shows the effects of different formulas on the sleep time of infants. At baseline, the average total sleep time of infants in all groups was 15.1 ± 3.3 h, the total sleep time of infants in the formula feeding groups was longer than that of the breastfeeding group, and the sleep time of infants in the FGM group was longer than that of the BF and M groups (*p* < .05).

At mid‐term and outcome, the average total sleep time of infants in all groups was 13.1 ± 2.0 h and 12.6 ± 1.5 h, respectively, and there was no difference in the total sleep time of infants among the different feeding groups (*p* > .05).

### 3.5 Allergic condition

**FIGURE 6:**
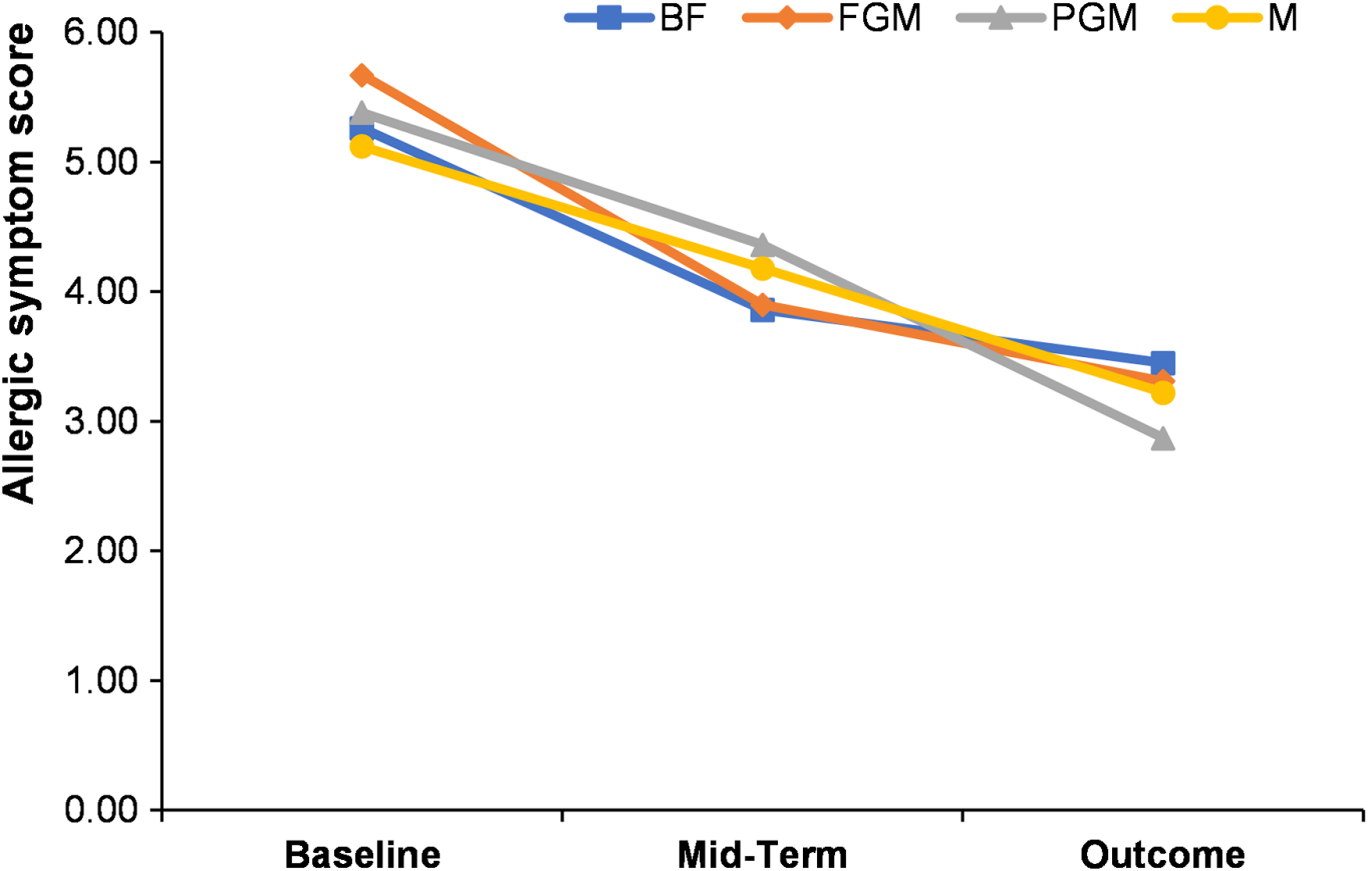
Effects of different formulas on allergy symptoms of infants

From baseline to outcome, the allergic symptom scores of the infants in each group showed a decreasing trend (Figure 6). The total allergic symptoms score of the BF group decreased from 5.26 ± 1.84 to 3.45 ± 1.42; the total score of the FGM group decreased from 5.67 ± 2.36 to 3.31 ± 1.66; the total score of the PGM group decreased from 5.38 ± 2.67 to 2.87 ± 1.97; and the total score of the M group decreased from 5.12 ± 2.29 to 3.22 ± 2.41. There were no differences in the total allergy symptom scores between the formula‐feeding and breastfeeding groups (*p* > .05).

### 3.6 The intestinal flora

*Bifidobacteria* are the main probiotics in the intestinal flora. After feeding for 6 months, the relative abundances of intestinal *Bifidobacteria* in the BF, FGM, PGM, and M groups were 34.1%, 31.1%, 25.6%, and 31.9%, respectively. The relative abundance of intestinal *Bifidobacteriaceae* in infants in the PGM group was lower than that in infants in the BF, FGM, and M groups (*p* < .05), and there were no differences in the relative abundance of intestinal *Bifidobacteriaceae* in infants in the FGM and M groups compared to that in the BF group (*p* > .05).

At baseline, infants in the formula‐feeding groups showed a higher α‐diversity index of the intestinal flora than those in the breastfeeding group (Table 4). After feeding for 6 months, the α‐diversity index of the breastfeeding group showed no apparent changes compared to the baseline; the *α*‐diversity index of infants fed formulas showed a decreasing trend. The variation range of the *α*‐diversity index in the formula‐feeding groups was higher than that in the breastfeeding group, and the variation range of the Chao1 index in the intestinal flora in the M group was higher than that in the BF and PGM groups (*p* < .05).

**TABLE 4:** The *α*‐diversity of the intestinal flora of infants in different groups at baseline and outcome [medium (P25, P75)]

	Chao1	Observed species	PD_whole_tree	Shannon	Simpson
**Baseline**					
BF	81.3 (59.9, 166.0)	60.0 (45.0, 101.0)	6.7 (5.0, 10.0)	2.4 (1.9, 3.1)	0.7 (0.6, 0.8)
FGM	134.6 (69.5, 234.9)^a^	69.0 (53.0, 147.0)	6.8 (5.2, 12.5)	2.6 (2.0, 3.1)	0.7 (0.6, 0.8)
PGM	115.4 (82.5, 187.4)^a^	69.0 (59.0, 115.5)^a^	7.5 (5.4, 10.9)	2.5 (2.0, 2.9)	0.7 (0.6, 0.8)
M	139.4 (92.3, 261.6)^a^	90.0 (58.5, 168.0)^a^	9.1 (5.6, 15.0)	2.7 (2.2, 3.1)	0.7 (0.7, 0.8)
*p*	0.036	0.017	0.089	0.800	0.339
**Outcome**					
BF	82.5 (63.9, 95.0)	62.0 (53.0, 71.0)	5.5 (4.4, 6.3)	2.6 (2.1, 3.1)	0.7 (0.7, 0.8)
FGM	83.3 (67.2, 106.8)	61.0 (54.0, 78.0)	5.7 (5.0, 7.0)	2.7 (2.3, 3.1)	0.8 (0.7, 0.8)
PGM	93.0 (77.1, 114.7)	70.0 (58.5, 80.0)^a^	6.5 (5.5, 7.3) ^a^ ^,^ ^b^	2.7 (2.3, 3.3)	0.8 (0.7, 0.8)
M	90.1 (72.4, 108.4)	72.0 (53.5, 82.5) ^a^ ^,^ ^b^	6.4 (5.4, 7.5) ^a^ ^,^ ^b^	2.6 (2.2, 3.1)	0.7 (0.6, 0.8)
*p*	0.171	0.024	<0.001	0.309	0.922

*Note*: Nonparametric test.

^a^ Compared with the BF group, there was a significant difference;

^b^ Compared with the FGM group, there was a significant difference.

**FIGURE 7:**
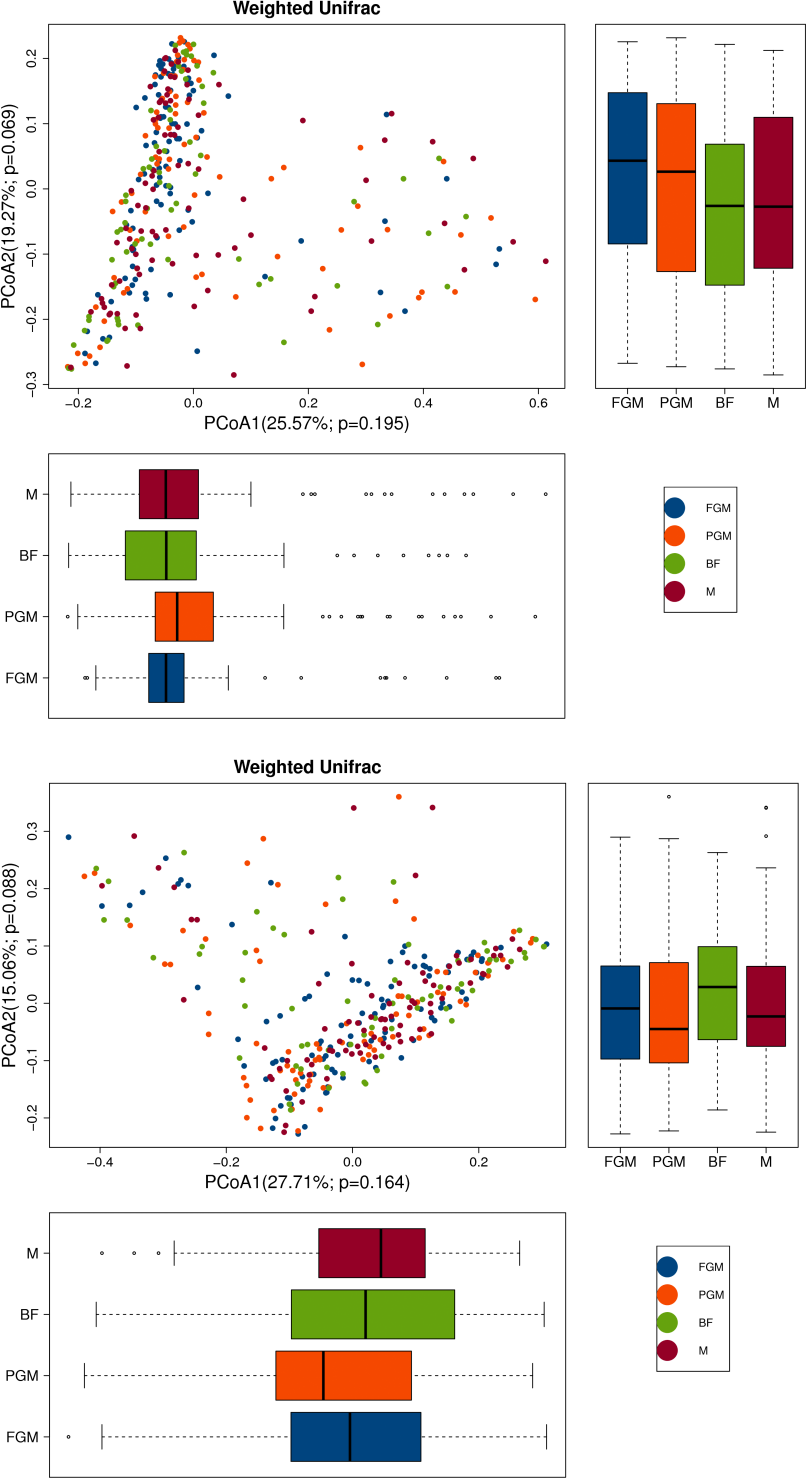
PCoA of the *β*‐diversity of intestinal flora among all groups at baseline (left) and outcome (right). Note: *p* value: Statistical reliability, less than .05, indicates a high reliability of the test. The horizontal and vertical coordinates represent the first and second principal coordinates, respectively, and the percentage represents the contribution rate of the corresponding principal coordinates to the sample difference.

As shown in Figure 7, there was no difference in the *β*‐diversity of intestinal flora between the formula‐feeding groups and the breastfeeding group at baseline and outcome (*p* > .05).

**FIGURE 8:**
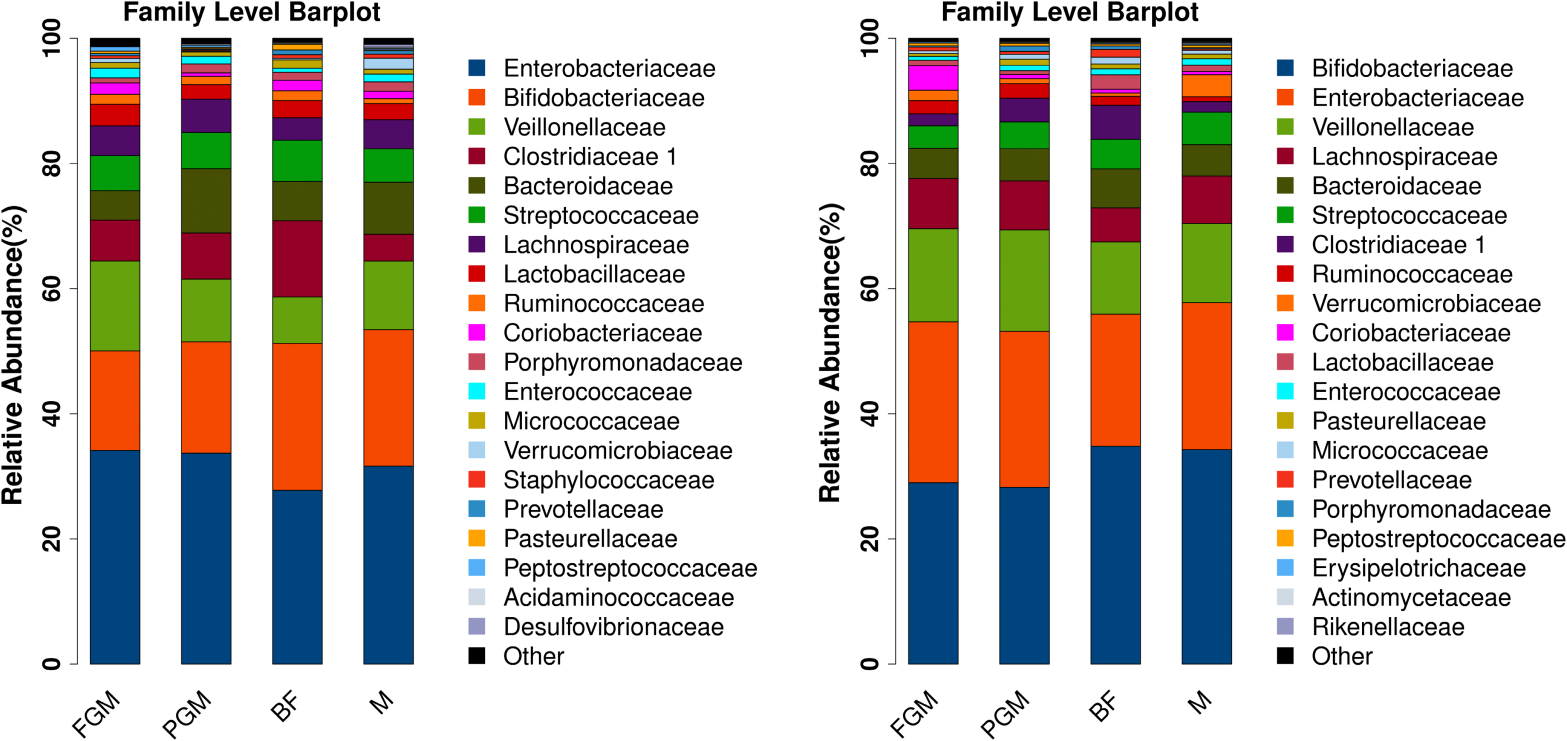
Comparison of the main intestinal flora composition of infants in different groups at baseline (left) and outcome (right) [family level]

At baseline and outcome, at the family level, there was a significantly high correlation between the main intestinal flora composition in the formula‐feeding and breastfeeding groups (Figure 8). The Spearman correlation coefficient between the main intestinal flora composition of infants in the FGM and BF groups was the highest (0.947 at baseline and 0.946 at outcome), followed by the PGM and M groups, but there was no statistically significant difference among the groups (*p* > .05).

## 4 DISCUSSION

At present, there are many kinds of infant formulas on the market, and consumers are still in the blind stage when choosing infant formulas. To address this issue, an increasing number of studies have analyzed and compared infant formulas. Xu et al. showed that the goat milk‐based formula is an appropriate alternative for infants who cannot be breastfed and are allergic to cow milk (Xu, Wang, et al., 2015). In an animal experiment, Xu et al. found that the goat milk‐based formula may play a role in the promotion of early growth, development, and immunity in weaned rats, especially in female rats (Xu, Wei, et al., 2015). Zhou et al. concluded that goat milk formula provided growth and nutritional outcomes in infants which did not differ from those provided with a standard whey‐based cow milk formula (Zhou et al., 2014). This study showed that compared with breastfeeding, all three formula feedings were able to meet the growth and developmental needs of infants, and there was no significant difference in the growth and development of infants with different formulas.

At the start of the study, baseline data suggested that breastfed infants had better growth and development than other groups; so early in life (0–3 months of age), breastfed infants probably had better growth and development than either artificial or mixed feeding. This shows that breast milk remains the best source of nutrition for infants. There were no significant differences in body length, weight, or head circumference between the artificial and breast‐fed groups as the infants got older. It was shown that feeding artificial formulas at the age of 4–9 months accelerated the physical development of infants and achieved an effect similar to that of breastfeeding. The study found that all four feeding methods were able to meet the needs of normal growth and development of infants (Bhandari & de Onis, 2007; WHO Multicentre Growth Reference Study Group, 2006), and the weight, length, and head circumference *Z* scores of infants in different feeding groups were within ±1 during different investigation periods, indicating that they were in a normal state of growth and development. This was in line with the research results of (Spalinger et al., 2017). The reason for this result may be that the nutritional compositions of breast milk are currently important references and matching standards for the formulation of infant formula, and with the continuous development of technology, the compositions of formulas are gradually similar to breast milk (Ahern et al., 2019; Heird, 2007; Hernell, 2011; Lönnerdal, 2008; Zhou et al., 2014).

At baseline, the prevalence rates of low weight, wasting, overweight, obesity, growth retardation, and total malnutrition of infants in the breastfeeding group were lower than those in the formula‐feeding groups. This further illustrated the importance of breastfeeding early in life (0–3 months old). After feeding for 3 months, compared with the baseline, the prevalence of all types of malnutrition in all groups showed a decreasing trend. After feeding for 6 months, the prevalence of low weight and wasting in each group continued to decrease, but the prevalence of overweight, obesity, and total malnutrition increased. It appeared that breastfeeding does not confer a protective effect against childhood overweight and obesity, which is consistent with some available studies. These studies suggest that breastfeeding does not reduce childhood obesity (Kramer, Matush, Vanilovich, Platt, Bogdanovich, Sevkovskaya, Dzikovich, Shishko, Collet, et al., 2007; Neutzling et al., 2009). Kramer et al. speculated that the previously reported beneficial effects may be the result of uncontrolled confounding and selection bias (Kramer, Matush, Vanilovich, Platt, Bogdanovich, Sevkovskaya, Dzikovich, Shishko, Collet, et al., 2007). However, some studies have also shown that breastfeeding has protective effects against overweight and obesity (Arenz et al., 2004a; Hunsberger, 2014; Owen et al., 2005). Controversy remains regarding whether and for how long breastfeeding is protective against childhood obesity, and further research is needed.

According to the health industry standard of the People's Republic of China “Sleep Hygiene Guidelines for Children 0‐5 Years Old (WS/T 579‐2017),” the recommended sleep time for infants aged 0–3 months and 4–11 months are 13–18 h and 12–16 h, respectively, and the WHO recommended sleep time for infants aged 0–3 months and 4–11 months are 14–17 h and 12–16 h (WHO, 2019), respectively, judging that the sleep time of infants in each group was within the normal range and that different feeding methods had no apparent effect on infant sleep time. At baseline, infants in the breastfed group slept less than those in the formula group, which may be related to multiple reasons, such as bed sharing and breastfeeding at night. Quillin et al. also demonstrated that breastfeeding was associated with less nighttime sleep in infants than formula‐feeding (Quillin, 1997). In another study with a larger sample size, Quillin et al. pointed out that breastfed infants have a shorter total daily sleep time than formula‐fed infants (Quillin & Glenn, 2004). The data from Huang et al. revealed that breastfeeding and sharing a bed may be related to insufficient sleep time in infants (Huang et al., 2016).

In terms of allergy symptoms, from baseline to outcome, it was found that the scores of allergy symptoms of infants in each group had a decreasing trend, which may be related to the tendency of allergy symptoms to decrease with the increase in infant age in months. There was no difference in the total allergy symptoms scores between the formula feeding and breastfeeding groups, and different feeding methods had no significant effect on the changes in allergic symptoms. The relationship between various infant feeding methods and the risk of allergy has been controversial (Goldsmith et al., 2016; Kramer, Matush, Vanilovich, Platt, Bogdanovich, Sevkovskaya, Dzikovich, Shishko, & Mazer, 2007; Mathias et al., 2019; Wegienka et al., 2006). Matthias et al. proved that mixed feeding could increase the risk of food allergy symptoms, but there was no significant difference in the incidence of allergies under different infant feeding patterns (Mathias et al., 2019). Wegienka G et al. concluded that breastfeeding without supplementary formula may increase the risk of allergies in children. Therefore, further studies are needed (Wegienka et al., 2006).

Analysis of the intestinal flora of infants showed that the relative abundance of intestinal *Bifidobacteria* in breastfed infants was higher than that in formula‐fed infants, which may be related to the composition of the breast milk. Bezirtzoglou et al. demonstrated that breastfed infants harbor fecal microbiota that is more than two times higher in the number of *Bifidobacterium* cells than formula‐fed infants (Bezirtzoglou et al., 2011). Breast milk differs from ruminant milk in that sialylated and fucosylated oligosaccharides (human milk oligosaccharides [HMOs]) are the third largest component. The unique human milk oligosaccharides and *Bifidus* factors in breast milk can promote the growth of *Bifidobacteria, Lactobacilli*, and *Bacteroides* while inhibiting the growth of other facultative anaerobes. Their presence in breast milk may explain why breastfed infants usually contain more of these bacteria in feces (Tannock et al., 2013). In addition, the relative abundance of intestinal *Bifidobacteriaceae* in infants of all groups increased with increasing age.

In early life (0–3 months), the number and diversity of intestinal flora of formula‐fed infants were higher than those of breastfed infants, which is consistent with the results of multiple studies (Bäckhed et al., 2015; Fan et al., 2014; Galazzo et al., 2020; Ho et al., 2018). Breastfeeding is conducive to maintaining the stability of the intestinal flora of infants. The main intestinal flora composition of infants fed different formulas was highly correlated with that of infants breastfed, and the Spearman correlation coefficient of the main intestinal flora composition of infants in the FGM and BF groups was the highest. The reason for this result may be that the kinetics of protein digestion of goat milk‐based infant formula are more comparable to those of human milk than those of cow milk‐based infant formula (Gallier et al., 2020; Maathuis et al., 2017).

This study has some limitations. First, to respect the choice of the infant's mother and improve their compliance, infants who were breastfed directly entered the control group, so this study was not a completely randomized controlled experiment; therefore, the evaluation of the intervention may be biased. Second, infants in the formula groups were artificially or mixed fed with more than 40% formulas. The higher proportion of mixed feeding may be an important reason why this study did not find much of a difference between breastfeeding and formula feeding. Some studies have concluded that infants who consume more formula have greater differences in growth than those who are breastfed (Hopkins et al., 2015; Huang et al., 2018; Kramer et al., 2004). Third, in terms of indicator selection, the indicators selected in this study were short‐term observation indicators. The shorter trial period may be another important reason why this study did not find much of a difference. Kramer et al. showed that breastfeeding children have similar growth rates as nonbreastfed children when only short‐term benefits are considered (Kramer & Kakuma, 2004). Regarding the long‐term consequences of breastfeeding, research suggests that infant feeding may influence the development of noncommunicable diseases in adulthood. Breastfeeding decreases the risk of obesity (Arenz et al., 2004b; Owen et al., 2005; Yan et al., 2014), diabetes (Bernardo & Cesar, 2013), and high blood pressure (Horta et al., 2015). Fourth, due to the limited number of staff, those who participated in the experimental grouping were also involved in data analysis, and the outcome assessors were not blinded, although the researchers maintained an objective and fair attitude.

With the rapid development of science and technology, the ingredients of formulas are getting closer to those of breast milk, which can gradually meet the needs of infant growth and development. However, further studies are needed to determine whether other risks are associated with formulas. More investigations are also required to determine the long‐term impact of formula feeding on infant health to illustrate the correlation between the impact on infant health and diseases.

## 5 CONCLUSIONS

This study showed that compared with breastfeeding, several formula feedings were able to meet the growth and developmental needs of infants, and within 6 months of feeding, there were no significant differences in the growth and development, allergic symptoms, and intestinal flora of the infants among the groups.

## FUNDING INFORMATION

This work was supported by Ausnutria Hyproca Nutrition. Ausnutria Hyproca Nutrition had no role in the design, analysis, or writing of this article.

## CONFLICT OF INTEREST

Craig‐Kui Xie and Yanmei Hou are employees of Ausnutria Hyproca Nutrition and only provided milk powder sample resources. All other authors have no conflicts of interest to declare.

## ETHICAL REVIEW

This study was approved by the Peking University Medical Ethics Committee (approval no: IRB00001052‐16037).

## INFORMED CONSENT

The mother or other guardian of each participant fully understood the relevant content of this study and signed an informed consent form.

## Supporting information


Appendix A

## Data Availability

The data presented in this study are available on request from the corresponding author. The data are not publicly available due to privacy.
