# Supplementary material for: Comparison of the effects of different infant formulas on the growth and development and intestinal flora of infants
Source: Food Sci Nutr. 2022 Dec 7;11(2):1113–26. doi: 10.1002/fsn3.3149 (PMC9922136; doi:10.1002/fsn3.3149)
Supplement: Supplementary file 1 — Appendix A [file FSN3-11-1113-s001.docx]

**Appendix A**

| Table Comparison of main nutrients of formula milk powder used in this study | | | | |
| --- | --- | --- | --- | --- |
| Main nutrients(/100kJ) | Full goat milk protein formula(FGM) | | Partial goat milk protein formula(PGM) | Milk formula(M) |
| Energy, kJ/100mL | | 285 | 307* | 275 |
| Protein, g | | 0.55 | 0.57 | 0.55 |
| Fat, g | | 1.24 | 1.24 | 1.24 |
| Carbohydrate, g | | 2.6 | 2.6 | 2.57 |
| Minerals | |  |  |  |
| Ca, mg | | 16 | 21 | 19.5 |
| P, mg | | 10.4 | 12 | 11 |
| K, mg | | 18 | 21 | 21.9 |
| Cl, mg | | 15 | 16 | 19 |
| Mg, mg | | 1.5 | 1.6 | 2.04 |
| Fe, mg | | 0.14 | 0.18 | 0.28 |
| Zn, mg | | 0.15 | 0.18 | 0.17 |
| Cu, μg | | 10.6 | 14.3 | 15.8 |
| Vitamins | |  |  |  |
| Vitamin A, μgRAE | | 17.5 | 21 | 27 |
| Vitamin D, μg | | 0.32 | 0.36 | 0.43 |
| Vitamin E, mg α-TE | | 0.29 | 0.19 | 0.51 |
| Vitamin B1, μg | | 17.6 | 26 | 20 |
| Vitamin B2, μg | | 40 | 31 | 62 |
| Vitamin B6, μg | | 9 | 14.3 | 17.0 |
| Vitamin B12, μg | | 0.033 | 0.04 | 0.136 |
| Niacin, μg | | 88 | 119 | 225 |
| Pantothenic acid, μg | | 120 | 167 | 170 |
| Biotin, μg | | 0.6 | 0.6 | 1.1 |
| Folic acid, μg | | 3.1 | 3.3 | 8.0 |
| Vitamin C, mg | | 3.1 | 3.3 | 4.3 |
| Others | |  |  |  |
| Taurine, mg | | 1.3 | 1.2 | 1.4 |
| L-carnitine, mg | | 0.38 | / | 0.5 |
| Nucleotide, mg | | 1.7 | 0.9 | 2.08 |

**Appendix B**

Table Infant sleep assessment scale

| 1. Infant sleeping arrangement:   a. Infant crib in a separate room b. Infant crib in parents’ room c. In parents' bed d. Infant crib in room with sibling e. In another family member's bed | | | | | |  |
| --- | --- | --- | --- | --- | --- | --- |
| 1. The most common sleeping position adopted by infants:   a. On his/her belly b. On his/her side c. On his/her back | | | | | |  |
| 1. Infant sleep time during the NIGHT(From 7:00 p.m. to 7:00 a.m.): hours | | | | | |  |
| 1. Infant sleep time during the DAY(From 7:00 a.m. to 7:00 p.m.): hours   Daytime naps:_________ | | | | | |  |
| 1. The number of times the infant wakes up from sleep at night is:   a. No b. 1 times c. 2 times d. 3 times or more | | | | | |  |
| 1. Time for infant to wake up during the night (From 10:00 p.m. to 7:00 a.m.): _________hours | | | | | |  |
| 1. How long does it usually take infant to fall asleep at night: hours minutes | | | | | |  |
| 1. Infant usually under what circumstances would fall asleep:   a. While feeding b. Being rocked c. Being held d. In bed alone e. In bed near parent | | | | | |  |
| 1. When does your baby usually fall asleep for the night: hours minutes | | | | | |  |
| 1. In the past month, does your baby has any of the following symptoms during sleep? (Please tick √)   a. None b. 1-2 times/week c. 3 times/week d. 4-5 times/week e. 6-7 times/week | | | | | |  |
| (1)Sleepiness during the day and wakefulness at night | a | b | c | d | e | |
| (2)Fall asleep early, even at dusk | a | b | c | d | e | |
| (3)Sleeping with mouth open | a | b | c | d | e | |
| (4)Breathing loudly while sleeping | a | b | c | d | e | |
| (5)Grind teeth while sleeping | a | b | c | d | e | |
| (6)Talking in sleep | a | b | c | d | e | |
| (7)Once mild stimulation in sleep just wake up | a | b | c | d | e | |
| (8)Crying and screaming in his sleep | a | b | c | d | e | |
| (9) limb twitches in sleep | a | b | c | d | e | |

**Appendix C**

Table Scoring criteria for infant allergy symptoms

| Symptom | Frequency | | | | Score |
| --- | --- | --- | --- | --- | --- |
| Crying | | ≤1 h/day | | | 0 |
|  |  | 1-1.5 h/day | | | 1 |
|  |  | 1.5-2 h/day | | | 2 |
|  |  | 2-3 h/day | | | 3 |
|  |  | 3-4 h/day | | | 4 |
|  |  | 4-5 h/day | | | 5 |
|  |  | ≥5 h/day | | | 6 |
| Regurgitation | | 0-2 episodes/day | | | 0 |
|  |  | ≥3 to ≤5 of small volume | | | 1 |
|  | | >5 episodes of >1 coffee spoon 20mL) | | | 2 |
|  |  | >5 episodes of ± half of the feed in < half of the feeds | | | 3 |
|  |  | Continuous regurgitations of small volumes >30 min after each feed | | | 4 |
|  |  | Regurgitation of half to complete volume of a feed in at least half of the feeds | | | 5 |
|  |  | Regurgitation of the complete feed after each feeding | | | 6 |
| Stools  (Bristol scale) | | hard stools | | | 4 |
|  |  | normal stools | | | 0 |
|  |  | soft stool | | | 2 |
|  |  | liquid stool, if unrelated to infection | | | 4 |
|  |  | watery stools | | | 6 |
| Skin symptoms | | Allergic eczema of head, neck and trunk | | Absent | 0 |
|  |  |  |  | Mild | 1 |
|  |  |  |  | Moderate | 2 |
|  |  |  |  | Severe | 3 |
|  |  | Allergic eczema of arms、hands、legs and feet | | Absent | 0 |
|  |  |  |  | Mild | 1 |
|  |  |  |  | Moderate | 2 |
|  |  |  |  | Severe | 3 |
|  |  | Urticaria | | No | 0 |
|  |  |  |  | Yes | 6 |
| Respiratory symptoms | | | No respiratory symptoms | | 0 |
|  |  |  | Slight symptoms | | 1 |
|  |  |  | Mild symptoms | | 2 |
|  |  |  | Severe symptoms | | 3 |
